# Supplementary material for: Choice and impact of EQ-5D-5L value set in cost-utility analyses alongside multinational trials: Insights from PREFERABLE-EFFECT and CONVINCE
Source: PLoS One. 2026 Jun 1;21(6):e0350019. doi: 10.1371/journal.pone.0350019 (PMC13225624; doi:10.1371/journal.pone.0350019)
Supplement: S1 File — Supplementary Material EQ5D PLOS One. Contains Supplementary Table S1-S7 and Supplementary Figure S1-S2. (DOCX) [file pone.0350019.s001.docx]

**Supplementary Material of the following manuscript:** Choice and Impact of EQ-5D-5L Value Set in Cost-Utility Analyses Alongside Multinational Trials: Insights From PREFERABLE-EFFECT and CONVINCE

**Supplementary Table S1.** Number of participants per randomization group per country

|  | **PREFERABLE-EFFECT (n = 357)** | | **CONVINCE (n = 1360)** | |
| --- | --- | --- | --- | --- |
|  | Intervention  (n = 178) | Control  (n = 179) | Intervention  (n = 677) | Control  (n = 683) |
| The Netherlands | 45 | 46 | 28 | 28 |
| Germany | 44 | 45 | 114 | 109 |
| Spain | 27 | 27 | 157 | 158 |
| Sweden | 22 | 22 |  |  |
| Poland | 22 | 22 |  |  |
| Australia | 18 | 17 |  |  |
| Romania |  |  | 177 | 180 |
| Portugal |  |  | 69 | 68 |
| France |  |  | 63 | 63 |
| Hungary |  |  | 57 | 53 |
| The United Kingdom |  |  | 18 | 18 |

**Supplementary Table S2.** Predictors included in the multiple imputation

| **Predictors** | **Details PREFERABLE-EFFECT** | **Details CONVINCE** |
| --- | --- | --- |
| The same EQ-5D domain values at other timepoints | Baseline (T0) vs 3 months (T1) vs 6 months (T2) vs 9 months (T3) | Baseline (T0) vs 6 months (T1) vs 12 months (T2) vs 18 months (T3) vs 24 months (T4) |
| Treatment Center or Country | NL1 vs NL2 vs DE1 vs DE2 vs ES vs SE vs PL vs AU | NL vs DE vs FR vs GB vs HU vs RO vs ES vs PT |
| Intervention group | Exercise vs control group | High-flux hemodialysis vs High-dose hemodiafiltration |
| Age | Year of birth | Age at baseline |
| Sex | Female / Male | Female / Male |
| Reason for ending study | Death vs voluntary withdrawal of consent vs study termination vs lost to follow-up vs other | Death (yes / no) |
| Timepoint of death | Alive vs between T0 and T1 vs between T1 and T2 vs between T2 and T3 | Number of years alive during trial (max = 2 years) |
| Co-morbidities | No comorbidities vs 1 comorbidity vs >1 comorbidity | Presence of cardiovascular diseases vs coronary heart disease vs diabetes at baseline (yes / no) |
| Hospitalization days | Length of stay | Number of days in hospital since last time point (starting at T1) |
| Physical fatigue | EORTC-QLQ-FA12 at each time point |  |
| Line of treatment at baseline | 1^st^/2^nd^ vs 3^rd^ or higher |  |
| Disease free interval | "mBC de novo","=<24 months",">24 months" |  |
| Cancer progression | yes/no for each timepoint |  |
| Tumor receptor status | Triple negative vs HER2 positive vs HER2 negative and hormone receptor positive |  |
| Location of metastasis | Bone only vs non-visceral only vs mixed (non-)visceral |  |
| Outpatient visits | Number of visits |  |
| Day treatment | Number of visits |  |
| Dialysis vintage |  | Length of time on dialysis (months) |
| Previous kidney transplantation |  | Yes / No |
| Type of vascular access |  | Fistula vs Catheter vs Graft |

**Supplementary Table S3.** Overview of the proportion of patients on each dimension of all EQ-5D domains and last follow-up moment.

|  | PREFERABLE-EFFECT (n = 357) | | | CONVINCE (n = 1360) | | |
| --- | --- | --- | --- | --- | --- | --- |
|  | Baseline | 9 months follow-up | 9 months follow-up (imputed) | Baseline | 24 months follow-up | 24 months follow-up (imputed) |
| Mobility [%] |  |  |  |  |  |  |
| No problems | 66.6% | 60.6% | 55.5% | 48.5% | 45.6% | 39.2% |
| Slight problems | 20.5% | 20.8% | 21.9% | 20.0% | 20.9% | 20.7% |
| Moderate problems | 11.5% | 13.8% | 16.3% | 18.5% | 18.9% | 21.0% |
| Severe problems | 1.4% | 4.5% | 5.9% | 10.2% | 10.9% | 14.4% |
| Unable to | 0.0% | 0.4% | 0.4% | 2.7% | 3.7% | 4.6% |
| *Missing (n)* | *(1)* | *(88)* | *-* | *(136)* | *(605)* | *-* |
| Self-care [%] |  |  |  |  |  |  |
| No problems | 93.0% | 85.1% | 81.6% | 79.1% | 72.6% | 65.4% |
| Slight problems | 6.5% | 11.9% | 14.9% | 9.7% | 11.6% | 13.7% |
| Moderate problems | 0.3% | 1.5% | 1.5% | 6.6% | 8.1% | 10.7% |
| Severe problems | 0.3% | 1.5% | 2.1% | 3.2% | 5.2% | 7.0% |
| Unable to | 0.0% | 0.0% | 0.0% | 1.5% | 2.5% | 3.2% |
| *Missing (n)* | *(1)* | *(88)* | *-* | *(128)* | *(604)* | *-* |
| Usual activities [%] |  |  |  |  |  |  |
| No problems | 55.6% | 48.7% | 45.9% | 49.5% | 46.2% | 39.8% |
| Slight problems | 29.8% | 30.9% | 32.0% | 26.2% | 23.5% | 23.7% |
| Moderate problems | 12.1% | 13.4% | 14.3% | 15.5% | 18.6% | 21.3% |
| Severe problems | 2.2% | 6.7% | 7.3% | 5.4% | 7.0% | 9.3% |
| Unable to | 0.3% | 0.4% | 0.5% | 3.4% | 4.7% | 5.9% |
| *Missing (n)* | *(1)* | *(88)* | *-* | *(127)* | *(602)* | *-* |
| Pain/ discomfort [%] |  |  |  |  |  |  |
| None | 33.4% | 30.1% | 28.0% | 37.4% | 37.3% | 32.8% |
| Slight | 44.4% | 40.1% | 40.6% | 34.9% | 31.8% | 31.4% |
| Moderate | 19.4% | 21.9% | 22.7% | 19.6% | 21.6% | 24.1% |
| Severe | 2.8% | 7.4% | 8.3% | 7.1% | 7.8% | 9.8% |
| Extreme | 0.0% | 0.4% | 0.4% | 0.9% | 1.6% | 1.9% |
| *Missing (n)* | *(1)* | *(88)* | *-* | *(123)* | *(599)* | *-* |
| Anxiety/depression [%] |  |  |  |  |  |  |
| None | 53.7% | 49.4% | 45.4% | 61.6% | 64.6% | 60.2% |
| Slight | 33.7% | 33.5% | 34.7% | 23.6% | 20.4% | 21.7% |
| Moderate | 9.3% | 11.2% | 12.8% | 11.1% | 11.1% | 13.1% |
| Severe | 2.8% | 5.2% | 6.2% | 2.6% | 3.0% | 4.0% |
| Extreme | 0.6% | 0.7% | 0.9% | 1.1% | 0.8% | 1.0% |
| *Missing (n)* | *(1)* | *(88)* | *-* | *(130)* | *(602)* | *-* |

**Supplementary Figure S1.** Violin plots with box plots of Quality-Adjusted Life Years of complete patient population using different value sets^1^

**
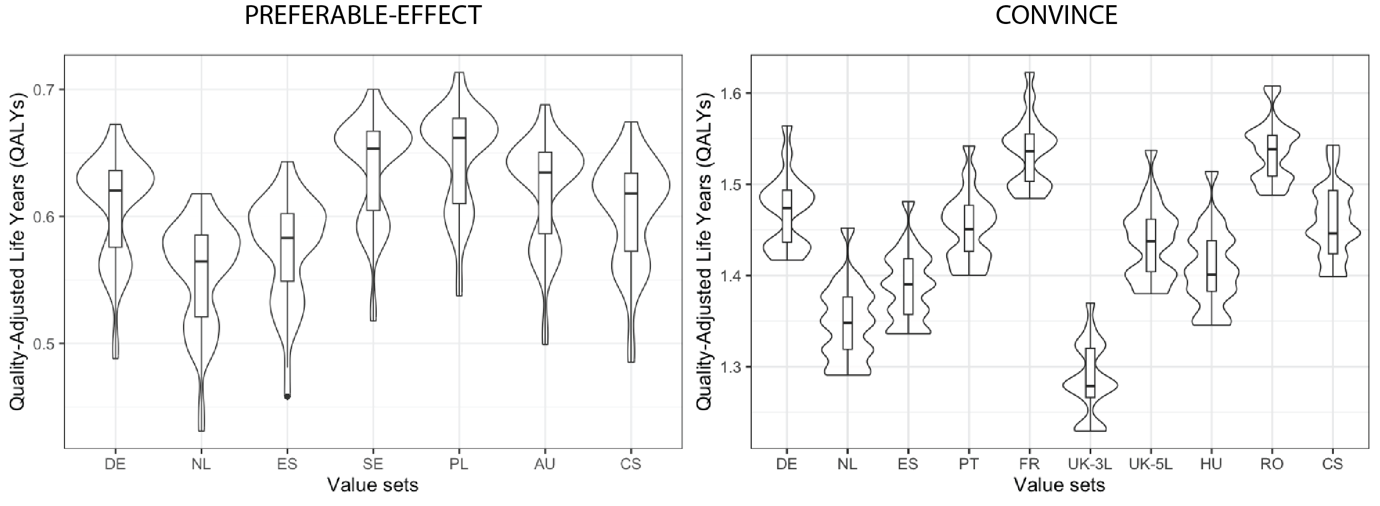
**DE, Germany; NL, the Netherlands; ES, Spain; SE, Sweden; PL, Poland; AU, Australia; CS, country-specific; PT, Portugal; FR, France; UK^1^, United Kingdom; HU, Hungary; RO, Romania

^1^ For the UK, the crosswalk version of the EQ-5D-3L value set was applied using the index values at the EuroQol website, following the national guideline. As an additional analysis, we also applied the 2026 EQ-5D-5L UK value set, although this is not officially adopted for national use at time of writing. For the country-specific value set, the EQ-5D-3L crosswalk value is used.

**Supplementary Figure S2.** Violin plots with box plots of utility scores per year alive of complete patient population using different value sets^1^

**
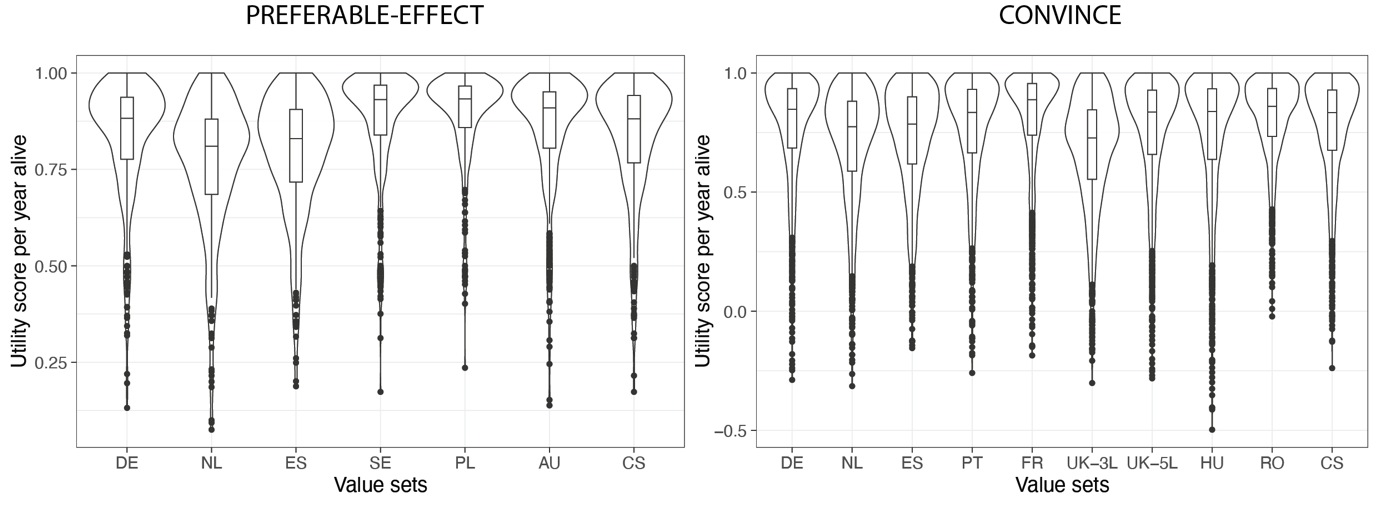
**

DE, Germany; NL, the Netherlands; ES, Spain; SE, Sweden; PL, Poland; AU, Australia; CS, country-specific; PT, Portugal; FR, France; UK^1^, United Kingdom; HU, Hungary; RO, Romania

^1^ For the UK, the crosswalk version of the EQ-5D-3L value set was applied using the index values at the EuroQol website, following the national guideline. As an additional analysis, we also applied the 2026 EQ-5D-5L UK value set, although this is not officially adopted for national use at time of writing. For the country-specific value set, the EQ-5D-3L crosswalk value is used.

**Supplementary Table S4.** Mean utility alive scores with the country-specific value set and the German value set

| **Mean utility alive** | **DE patients** | **NL patients** | **ES patients** | **SE patients** | **PL patients** | **AU patients** |  |  |
| --- | --- | --- | --- | --- | --- | --- | --- | --- |
| ***PREFERABLE*** |  |  |  |  |  |  |  |  |
| *N* | 89 | 91 | 54 | 44 | 44 | 35 |  |  |
| DE value set | 0.812 | 0.856 | 0.881 | 0.783 | 0.81 | 0.851 |  |  |
| NL value set | 0.732 | 0.791 | 0.828 | 0.709 | 0.723 | 0.774 |  |  |
| ES value set | 0.765 | 0.812 | 0.844 | 0.744 | 0.752 | 0.807 |  |  |
| SE value set | 0.855 | 0.897 | 0.911 | 0.834 | 0.858 | 0.896 |  |  |
| PL value set | 0.874 | 0.903 | 0.911 | 0.849 | 0.885 | 0.909 |  |  |
| AU value set | 0.833 | 0.872 | 0.888 | 0.801 | 0.832 | 0.871 |  |  |
| CS value set | 0.812 | 0.791 | 0.844 | 0.834 | 0.885 | 0.871 |  |  |
| **Difference highest-lowest** | **0.142** | **0.112** | **0.083** | **0.140** | **0.162** | **0.135** |  |  |
| **Mean utility alive** | **DE patients** | **NL patients** | **ES patients** | **PT patients** | **FR patients** | **UK patients** | **HU patients** | **RO patients** |
| ***CONVINCE*** |  |  |  |  |  |  |  |  |
| *N* | 223 | 56 | 315 | 137 | 126 | 36 | 110 | 357 |
| DE value set | 0.734 | 0.748 | 0.760 | 0.819 | 0.774 | 0.699 | 0.835 | 0.776 |
| NL value set | 0.664 | 0.679 | 0.692 | 0.756 | 0.706 | 0.626 | 0.771 | 0.715 |
| ES value set | 0.688 | 0.700 | 0.717 | 0.774 | 0.727 | 0.657 | 0.783 | 0.737 |
| PT value set | 0.724 | 0.736 | 0.749 | 0.807 | 0.765 | 0.691 | 0.821 | 0.770 |
| FR value set | 0.771 | 0.785 | 0.795 | 0.847 | 0.808 | 0.734 | 0.865 | 0.812 |
| UK 3L value set^1^ | 0.626 | 0.633 | 0.657 | 0.718 | 0.677 | 0.589 | 0.738 | 0.69 |
| UK 5L value set^1^ | 0.714 | 0.728 | 0.741 | 0.802 | 0.755 | 0.674 | 0.820 | 0.760 |
| HU value set | 0.689 | 0.708 | 0.724 | 0.788 | 0.736 | 0.643 | 0.811 | 0.746 |
| RO value set | 0.780 | 0.790 | 0.798 | 0.842 | 0.815 | 0.758 | 0.85 | 0.811 |
| CS value set | 0.734 | 0.679 | 0.717 | 0.807 | 0.808 | 0.589 | 0.811 | 0.811 |
| **Difference highest-lowest** | **0.154** | **0.157** | **0.141** | **0.129** | **0.138** | **0.169** | **0.127** | **0.122** |

DE, Germany; NL, the Netherlands; ES, Spain; SE, Sweden; PL, Poland; AU, Australia; CS, country-specific; PT, Portugal; FR, France; UK, United Kingdom; HU, Hungary; RO, Romania

^1^ For the UK, the crosswalk version of the EQ-5D-3L value set was applied using the index values at the EuroQol website, following the national guideline. As an additional analysis, we also applied the 2026 EQ-5D-5L UK value set, although this is not officially adopted for national use at time of writing. For the country-specific value set, the EQ-5D-3L crosswalk value is used.

**Supplementary Table S5.** Mean utility alive scores of the complete patient population per randomization group using different value sets

|  | **Mean utility alive (intervention)** | **Mean utility alive (control)** | **Difference** |
| --- | --- | --- | --- |
| **PREFERABLE EFFECT** |  |  |  |
| DE value set | 0.850 | 0.843 | 0.007 |
| NL value set | 0.781 | 0.766 | 0.015 |
| ES value set | 0.809 | 0.792 | 0.017 |
| SE value set | 0.891 | 0.887 | 0.004 |
| PL value set | 0.905 | 0.898 | 0.008 |
| AU value set | 0.868 | 0.86 | 0.008 |
| CS value set | 0.846 | 0.837 | 0.010 |
| **Difference**  **highest-lowest** | **0.124** | **0.132** | **0.013** |
| **CONVINCE** |  |  |  |
| DE value set | 0.802 | 0.782 | 0.020 |
| NL value set | 0.736 | 0.715 | 0.021 |
| ES value set | 0.757 | 0.738 | 0.019 |
| PT value set | 0.791 | 0.774 | 0.017 |
| FR value set | 0.835 | 0.818 | 0.017 |
| UK 3L value set  UK 5L value set | 0.701  0.784 | 0.684  0.763 | 0.017  0.021 |
| HU value set | 0.768 | 0.748 | 0.020 |
| RO value set | 0.834 | 0.821 | 0.013 |
| CS value set | 0.793 | 0.778 | 0.015 |
| **Difference**  **highest-lowest** | **0.134** | **0.137** | **0.008** |

DE, Germany; NL, the Netherlands; ES, Spain; SE, Sweden; PL, Poland; AU, Australia; CS, country-specific; PT, Portugal; FR, France; UK, United Kingdom; HU, Hungary; RO, Romania

^1^ For the UK, the crosswalk version of the EQ-5D-3L value set was applied using the index values at the EuroQol website, following the national guideline. As an additional analysis, we also applied the 2026 EQ-5D-5L UK value set, although this is not officially adopted for national use at time of writing. For the country-specific value set, the EQ-5D-3L crosswalk value is used.

**Supplementary Table S6.** Difference in utility value alive between intervention and control across imputation scenarios and value sets – PREFERABLE-EFFECT

| **Scenarios** | **NL** | **DE** | **ES** | **SE** | **PL** | **AU** | **CS** | **Min** | **Max** | **Mean** | **Difference (Max - Min)** |
| --- | --- | --- | --- | --- | --- | --- | --- | --- | --- | --- | --- |
| **"First imputation, then value sets"** | | | |  |  |  |  |  |  |  |  |
| **Seed: 123** | -0.015 | -0.007 | -0.017 | -0.004 | -0.008 | -0.008 | -0.010 | -0.017 | -0.004 | -0.010 | **0.013** |
| **Seed: 234** | -0.016 | -0.008 | -0.018 | -0.005 | -0.008 | -0.009 | -0.010 | -0.018 | -0.005 | -0.011 | **0.013** |
| **Seed: 48959** | -0.014 | -0.007 | -0.016 | -0.004 | -0.007 | -0.007 | -0.009 | -0.016 | -0.004 | -0.009 | **0.012** |
| **"First value sets, then imputation"** | | | |  |  |  |  |  |  |  |  |
| **Seed: 123** | -0.019 | -0.007 | -0.015 | -0.004 | -0.009 | -0.004 | -0.013 | -0.019 | -0.004 | -0.010 | **0.016** |
| **Seed: 234** | -0.015 | -0.005 | -0.014 | -0.002 | -0.008 | -0.005 | -0.011 | -0.015 | -0.002 | -0.008 | **0.013** |
| **Seed: 48959** | -0.013 | -0.004 | -0.013 | -0.002 | -0.008 | -0.009 | -0.013 | -0.013 | -0.002 | -0.009 | **0.011** |
| **Min** | -0.019 | -0.011 | -0.020 | -0.008 | -0.009 | -0.013 | -0.015 |  |  |  |  |
| **Max** | -0.013 | -0.004 | -0.013 | -0.002 | -0.006 | -0.004 | -0.008 |  |  |  |  |
| **Mean** | -0.016 | -0.007 | -0.017 | -0.004 | -0.008 | -0.008 | -0.011 |  |  |  |  |
| **Difference within value set (Max-Min)** | 0.006 | 0.004 | 0.005 | 0.003 | 0.002 | 0.005 | 0.004 |  |  | **0.004** |  |
| **MEAN** |  |  |  |  |  |  |  |  |  |  | **0.013** |

DE, Germany; NL, the Netherlands; ES, Spain; SE, Sweden; PL, Poland; AU, Australia; CS, country-specific

**Supplementary Table S7.** Difference in utility value alive between intervention and control across imputation scenarios and value sets – CONVINCE

| **Scenarios** | **NL** | **DE** | **FR** | **UK-3L** | **ES** | **PT** | **HU** | **RO** | **CS** | **Min** | **Max** | **Mean** | **Δ (Max - Min)** |
| --- | --- | --- | --- | --- | --- | --- | --- | --- | --- | --- | --- | --- | --- |
| **"First imputation, then value sets"** | | | |  |  |  |  |  |  |  |  |  |  |
| **Seed: 123** | 0.021 | 0.020 | 0.017 | 0.017 | 0.019 | 0.017 | 0.020 | 0.013 | 0.015 | 0.021 | 0.013 | 0.018 | **0.008** |
| **Seed: 234** | 0.022 | 0.021 | 0.018 | 0.019 | 0.020 | 0.017 | 0.021 | 0.014 | 0.016 | 0.022 | 0.014 | 0.018 | **0.008** |
| **Seed: 48959** | 0.021 | 0.020 | 0.017 | 0.017 | 0.019 | 0.017 | 0.020 | 0.013 | 0.015 | 0.021 | 0.013 | 0.018 | **0.009** |
| **First value set, then imputation** | | | |  |  |  |  |  |  |  |  |  |  |
| **Seed: 123** | 0.021 | 0.021 | 0.017 | 0.022 | 0.020 | 0.018 | 0.020 | 0.014 | 0.015 | 0.022 | 0.014 | 0.019 | **0.007** |
| **Seed: 234** | 0.022 | 0.023 | 0.017 | 0.020 | 0.021 | 0.018 | 0.021 | 0.015 | 0.017 | 0.023 | 0.015 | 0.019 | **0.008** |
| **Seed: 48959** | 0.024 | 0.023 | 0.019 | 0.022 | 0.021 | 0.019 | 0.022 | 0.014 | 0.018 | 0.024 | 0.014 | 0.020 | **0.010** |
|  |  |  |  |  |  |  |  |  |  |  |  |  |  |
| **Min** | 0.021 | 0.020 | 0.017 | 0.017 | 0.019 | 0.017 | 0.020 | 0.013 | 0.015 |  |  |  |  |
| **Max** | 0.024 | 0.023 | 0.019 | 0.022 | 0.021 | 0.019 | 0.022 | 0.015 | 0.018 |  |  |  |  |
| **Mean** | 0.022 | 0.022 | 0.017 | 0.020 | 0.020 | 0.018 | 0.021 | 0.014 | 0.016 |  |  |  |  |
| **Difference**  **within value**  **set (Max-Min)** | 0.003 | 0.003 | 0.002 | 0.005 | 0.002 | 0.003 | 0.003 | 0.002 | 0.003 |  |  | 0.003 |  |
| **MEAN** |  |  |  |  |  |  |  |  |  |  |  |  | **0.008** |

DE, Germany; NL, the Netherlands; ES, Spain; CS, country-specific; PT, Portugal; FR, France; UK, United Kingdom; HU, Hungary; RO, Romania
