# Supplementary material for: Choice and impact of EQ-5D-5L value set in cost-utility analyses alongside multinational trials: Insights from PREFERABLE-EFFECT and CONVINCE
Source: PLoS One. 2026 Jun 1;21(6):e0350019. doi: 10.1371/journal.pone.0350019 (PMC13225624; doi:10.1371/journal.pone.0350019)
Supplement: Attachment — Submitted filename: paper review_plos one.docx [file pone.0350019.s002.docx]

Dear author:

I greatly enjoyed reading this manuscript and learned a lot from your analysis. The topic is highly relevant and the methodological approach is carefully conducted. I only have several minor comments that may help improve clarity and readability for a broader audience.

Major comments:

1. I suggest that the research gap be articulated more explicitly in the objective section. While the study aim is clearly stated, it would strengthen the abstract to briefly clarify what remains uncertain in the existing literature and how this study addresses that gap.

2. The two trials (PREFERABLE-EFFECT and CONVINCE) involve different patient populations and disease contexts. It would be helpful to briefly clarify whether differences in demographics or baseline disease severity between these trials may influence the observed variation in utility estimates across value sets.

3. The manuscript states: “Health economic evaluation guidelines recommend using the value set of the jurisdiction of interest. However, there is currently no guideline on which value set(s) should be used…” This statement could benefit from clarification. Since national guidelines do provide recommendations for jurisdiction-specific analyses, it would be helpful to more clearly explain the gap that exists specifically in the context of pooled multinational analyses.

4. The statement that “there is no standard practice for estimating utilities” could be further elaborated. I suggest that the authors briefly describe how pooled multinational analyses are currently conducted in practice to provide clearer context.

5. The manuscript mentions that the study includes patients with different chronic conditions. It may be helpful to clarify whether differences in disease severity or symptom burden were considered as potential modifiers of the impact of value set choice.

**6.** The sentence “Utility scores of the full sample were calculated with the value sets of each included country, and with the country-specific value set of each participant” is somewhat unclear. Could the authors please clarify exactly how these two approaches differ in practice? Specifically, does “value sets of each included country” mean that the entire pooled sample was re-valued multiple times using each country's tariff separately?

7. It would be helpful if the authors could further justify the selection of the PREFERABLE-EFFECT and CONVINCE trials. As the two studies involve distinct patient populations (one predominantly female, one predominantly male), it may be useful to clarify the rationales.

8. “The imputed datasets were analyzed using Rubin’s rules” could you please provide additional explanations on Rubin’s rule?

9. I am slightly confused as to whether the authors applied both the crosswalk version of the EQ-5D-3L and the EQ-5D-5L UK value set in the main analyses.

10. In Table 2, the number of respondents differs substantially across countries, and the variation appears quite large. Have the authors examined whether these differences in sample size may influence the estimated utility values?

Minor comments

1 in Line 126, the abbreviations of ‘Netherlands, Germany, Spain,’ are lacked.
