# Supplementary material for: Choice and impact of EQ-5D-5L value set in cost-utility analyses alongside multinational trials: Insights from PREFERABLE-EFFECT and CONVINCE
Source: PLoS One. 2026 Jun 1;21(6):e0350019. doi: 10.1371/journal.pone.0350019 (PMC13225624; doi:10.1371/journal.pone.0350019)
Supplement: Attachment — Submitted filename: Review comments.docx [file pone.0350019.s003.docx]

Thank you for the opportunity to review this manuscript.

This study investigates how the choice of EQ-5D-5L value sets affect cost-utility outcomes using two multinational trials. The authors compare utility scores and QALY estimated using a single value set against those derived from country specific value sets. Below, I provide some comments that I believe will improve the quality of the manuscript.

- Line 83-84: The introduction seems to have a contradicting problem statement. Specifically, the following two sentences “Health economic evaluation guidelines recommend using the value set of the jurisdiction of interest. However, there is currently no guideline on which value set(s) should be used for these types of analyses to estimate utility scores.” I think it might be helpful for authors to clarify whether the gap lies in the absence of guidelines for multinational pooled analysis, or a broader absence of consensus. I believe clarifying this point is important as it is the core rationale for the study.
- Line 95: The authors mention that “Oppong et al. (2010) found that the choice between European, country-specific, or UK value sets in country-specific sub-group analyses had little impact on cost-utility outcomes when using the EQ-5D-3L.” I think authors should discuss how the findings of this study relate to the findings of Oppong et al.
- To strengthen its policy relevance, I would suggest adding the European policy context and broader European HTA landscape. The recent EU HTA regulation has a direct implication for how pooled data should be handled.
- Missing data in CONVINCE is concerningly high. I wonder if the authors have some possible explanations for that.
- Lines 144-147, it is quite difficult to follow what is being imputed/calculated, I assume mainly due to the possibility of different terminologies being used across fields. It would be helpful if the authors consider using some mathematical formulas for clarity, which can then be put in the appendix.
- If the authors would like to keep the paper up to date, the UK value set for the EQ-5D-5L is now available (<https://doi.org/10.1016/j.jval.2026.03.008> )
