# Supplementary material for: Choice and impact of EQ-5D-5L value set in cost-utility analyses alongside multinational trials: Insights from PREFERABLE-EFFECT and CONVINCE
Source: PLoS One. 2026 Jun 1;21(6):e0350019. doi: 10.1371/journal.pone.0350019 (PMC13225624; doi:10.1371/journal.pone.0350019)
Supplement: Attachment — Submitted filename: Response to reviewers_EQ5D_PLOS-One.docx [file pone.0350019.s005.docx]

**Response to reviewers**

May 4, 2026

Manuscript ID: PONE-D-25-69177

Manuscript title: Choice and Impact of EQ-5D-5L Value Set in Cost-Utility Analyses Alongside Multinational Trials: Insights From PREFERABLE-EFFECT and CONVINCE

*Thank you for considering our paper for publication in PLOS One, and the opportunity to address the comments from the reviewers. We incorporated most of the feedback and agree that these improve the quality of our manuscript. The changes in the manuscript are highlighted with ‘track changes’. Please find our detailed responses on all comments below.*

*Kind regards,*

*On behalf of the authors,*

*Aniek Schouten*

Dear author:

I greatly enjoyed reading this manuscript and learned a lot from your analysis. The topic is highly relevant and the methodological approach is carefully conducted. I only have several minor comments that may help improve clarity and readability for a broader audience.

*Authors’ response*

*We thank the reviewer for the positive feedback. Below we answer the specific comments and agree that these improve the manuscript's clarity and readability.*

Major comments:

1. I suggest that the research gap be articulated more explicitly in the objective section. While the study aim is clearly stated, it would strengthen the abstract to briefly clarify what remains uncertain in the existing literature and how this study addresses that gap.

*Authors’ response*

*We agree with this comment. We have added the following to the objective section:*

**Multinational clinical trials are increasingly common in the European Economic Area, yet no guideline exists on which EQ-5D value set(s) should be used for health economic evaluations alongside multinational trials.** Either a single value set or country-specific value sets can be applied**, and previous studies have shown that the choice of value set can impact the estimated utility scores. For the EQ-5D-5L, the impact on European pooled cost-utility analyses has not been established.** This study evaluates the impact of EQ-5D-5L value sets on cost-utility outcomes in two multinational trials.

2. The two trials (PREFERABLE-EFFECT and CONVINCE) involve different patient populations and disease contexts. It would be helpful to briefly clarify whether differences in demographics or baseline disease severity between these trials may influence the observed variation in utility estimates across value sets.

*Authors’ response*

*We agree that the two trials are quite distinct, which is also the reason that we included the data of both studies. We know that the cost-utility outcomes of the trials are different, as these have been previously analyzed for both trials separately. For this reason, the aim of this manuscript was not to see whether there was a difference in utility estimates between the two studies. Rather, we wanted to see whether the choice of value set impacts utility estimates and cost-utility outcomes, and if this impact is consistent across different analyses and patient populations. We thus see it as a strength to include the different patient populations and disease contexts rather than as an obstacle.*

*We saw that despite the differences in studies, the impact on absolute utility scores was quite similar across the two studies. The maximum difference in utility scores per year alive was 0.127 and 0.133 for PREFERABLE-EFFECT and CONVINCE, respectively (Table 1). When comparing the impact of value sets within country subgroups, we found a maximum difference in baseline utility scores of -0.065 (PREFERABLE-EFFECT) and -0.069 (CONVINCE) for Dutch patients comparing the Dutch and the German value set, and -0.037 (PREFERABLE-EFFECT) and -0.043 (CONVINCE) for Spanish patients comparing the Spanish and German value set (Table 2). This indicates that despite differences in demographics or baseline disease severity, the impact on utility estimates as a result of choosing a different value set is consistent. We have added this clarification in the first paragraph of the discussion:*

These results suggest that cost-utility outcomes may be impacted by **the** choice of value set **in different patient populations.**

3. The manuscript states: “Health economic evaluation guidelines recommend using the value set of the jurisdiction of interest. However, there is currently no guideline on which value set(s) should be used…” This statement could benefit from clarification. Since national guidelines do provide recommendations for jurisdiction-specific analyses, it would be helpful to more clearly explain the gap that exists specifically in the context of pooled multinational analyses.

*Authors’ response*

*We agree with the suggestion for clarification. We have changed the sentence in the following way:*

Health economic evaluation guidelines recommend using the value set of the jurisdiction of interest **for single-country studies**.^11^ However, there is currently no guideline on which value set(s) should be used for **(pooled) multinational health economic evaluations**.

4. The statement that “there is no standard practice for estimating utilities” could be further elaborated. I suggest that the authors briefly describe how pooled multinational analyses are currently conducted in practice to provide clearer context.

*Authors’ response*

*We have rephrased the sentence following this statement to clarify that this was intended to describe how these analyses are currently conducted in practice:*

For pooled analyses, there is no standard practice for estimating utilities.^15^ **In practice**, a single value set is **most commonly** **applied**, often that of the UK as it was the first developed value set.^6,16^ An alternative is applying the country-specific value set of each participant. A single value set, reflecting the preferences of a larger region, has been proposed as solution,^17-20^ but this is currently not commonly adopted.^10^

5. The manuscript mentions that the study includes patients with different chronic conditions. It may be helpful to clarify whether differences in disease severity or symptom burden were considered as potential modifiers of the impact of value set choice.

*Authors’ response*

*See our answer to question 2.*

*Furthermore, for both studies, we included baseline utility estimates as control variable in the seemingly unrelated regression analyses to estimate the QALY difference. Our expectation is that the variation in QALY gains as a result of choice of value set (maximum difference of 0.007 for PREFERABLE-EFFECT and 0.013 for CONVINCE) is more impacted by the duration of follow-up and a difference in the impact of the intervention on mortality, rather than differences in demographics. Data was collected for 9 months for PREFERABLE-EFFECT, which means the maximum QALY that could be estimated for a participant over that period would be 0.75 (9 months at perfect health). For CONVINCE, data was collected for 24 months, which means that over this period the maximum estimated QALY of a participant could have been 2.00 (24 months in perfect health). This means the cumulative impact of a value set is expected to be higher for studies with a longer study follow-up, just like QALY gains are expected to be higher for studies with similar impact but with a longer time horizon.*

6. The sentence “Utility scores of the full sample were calculated with the value sets of each included country, and with the country-specific value set of each participant” is somewhat unclear. Could the authors please clarify exactly how these two approaches differ in practice? Specifically, does “value sets of each included country” mean that the entire pooled sample was re-valued multiple times using each country's tariff separately?

*Authors’ response*

*Indeed. We have clarified this sentence in the following way:*

Based on the imputed domains, utilities were **estimated multiple times** for the entire study population **by applying the** EQ-5D-5L value set of each included country of the study **separately**,^41-51^ as well as the appropriate country-specific value set per patient (CS).

7. It would be helpful if the authors could further justify the selection of the PREFERABLE-EFFECT and CONVINCE trials. As the two studies involve distinct patient populations (one predominantly female, one predominantly male), it may be useful to clarify the rationales.

*Authors’ response*

*We have added the following justification to the last paragraph of the introduction:*

**The two studies include distinct patient populations (patients with metastatic breast cancer and kidney failure), to assess whether the observed impact is consistent across different demographic groups.**

8. “The imputed datasets were analyzed using Rubin’s rules” could you please provide additional explanations on Rubin’s rule?

*Authors’ response*

*We have provided the following additional explanation:*

The imputed datasets were analyzed using Rubin’s rules**, a method that combines (pools) results from multiple imputed datasets and adjusts the standard error to reflect the uncertainty introduced by the missing data**.^40^

9. I am slightly confused as to whether the authors applied both the crosswalk version of the EQ-5D-3L and the EQ-5D-5L UK value set in the main analyses.

*Authors’ response*

*We applied the crosswalk version of the UK EQ-5D-3L, and not of the UK EQ-5D-5L value set. The crosswalk version of the EQ-5D-3L ‘translates’ the value set of the 3-level version of the EQ-5D to a value set that can be used for a 5-level EQ-5D questionnaire. Since the EQ-5D-5L value set is already directly applicable to the 5-level EQ-5D questionnaire, we did not need to apply a crosswalk version. We did apply both the EQ-5D-5L value set and the crosswalk version of the EQ-5D-3L value set. We included an analysis with this EQ-5D-3L crosswalk value set, as this is the version officially recommended by the UK national guideline, having rejected the use of the EQ-5D-5L value set (e.g., see* Sampson C. (2022). NICE and the EQ-5D-5L: Ten Years Trouble. *PharmacoEconomics - open, 6*(1), 5–8. <https://doi.org/10.1007/s41669-021-00315-1>*).*

*The other reviewer pointed out that a new EQ-5D-5L value set has been established and published for the UK in the last month (see* Rowen, D., Mukuria, C., Bray, N., Carlton, J., Longworth, L., Meads, D., ... & Yang, Y. (2026). A UK value set for the EQ-5D-5L. *Value in Health). As the National Institute for Health and Care Excellence (NICE) plans to adopt this version as the new value set, we have replaced the values of the rejected EQ-5D-5L version with the new value set (see* [*https://euroqol.org/new-eq-5d-5l-value-set-for-the-united-kingdom-published/*](https://euroqol.org/new-eq-5d-5l-value-set-for-the-united-kingdom-published/)*). Of note, this resulted in similar results compared to the previous version of the EQ-5D-5L value set.*

10. In Table 2, the number of respondents differs substantially across countries, and the variation appears quite large. Have the authors examined whether these differences in sample size may influence the estimated utility values?

*Authors’ response*

*We agree that the number of respondents per country are likely to influence the absolute value and uncertainty of the estimates presented in table 2. However, for this table we were mainly interested in seeing whether the order of the countries in terms of utility score (highest>lowest) changes, which is not dependent on sample size.*

*The differences in sample size of the included countries should not affect the results presented in the other tables and figures, as we look at the overall pooled results of all trial participants. These differences in number of patients per country are inherently related to the nature of multinational trials and are therefore real-world reflections.*

*If we wanted to estimate the cost-utility outcomes for each country separately, then indeed the differences in sample size are likely to influence results (not just for the estimated utility values, but also for costs), especially as statistical power is likely not sufficient to perform these subgroup analyses. For this reason, we focused on pooled outcomes in this manuscript and have not estimated the impact on cost-utility outcomes for each country separately.*

Minor comments

1 in Line 126, the abbreviations of ‘Netherlands, Germany, Spain,’ are lacked.

*Authors’ response*

*These abbreviations were left out as they were defined in the paragraph above. However, for consistency we have added the abbreviations as suggested.*

Thank you for the opportunity to review this manuscript.

This study investigates how the choice of EQ-5D-5L value sets affect cost-utility outcomes using two multinational trials. The authors compare utility scores and QALY estimated using a single value set against those derived from country specific value sets. Below, I provide some comments that I believe will improve the quality of the manuscript.

- Line 83-84: The introduction seems to have a contradicting problem statement. Specifically, the following two sentences “Health economic evaluation guidelines recommend using the value set of the jurisdiction of interest. However, there is currently no guideline on which value set(s) should be used for these types of analyses to estimate utility scores.” I think it might be helpful for authors to clarify whether the gap lies in the absence of guidelines for multinational pooled analysis, or a broader absence of consensus. I believe clarifying this point is important as it is the core rationale for the study.

*Authors’ response*

*We agree that this is unclear. We have changed the sentence in the following way:*

Health economic evaluation guidelines recommend using the value set of the jurisdiction of interest **for single-country studies**.^11^ However, there is currently no guideline on which value set(s) should be used for **(pooled) multinational health economic evaluations**.

- Line 95: The authors mention that “Oppong et al. (2010) found that the choice between European, country-specific, or UK value sets in country-specific sub-group analyses had little impact on cost-utility outcomes when using the EQ-5D-3L.” I think authors should discuss how the findings of this study relate to the findings of Oppong et al.

*Authors’ response*

*We agree. We have added the following in the 2nd paragraph of the discussion:*

**Similar to the findings of Oppong et al. (2011) and van Dongen et al. (2021), we found that** for the trial-based analyses, value sets that resulted in higher absolute utility scores did not directly lead to a higher or lower incremental difference between intervention and control.^16,23^

*And the 1st paragraph of the implications section in the discussion:*

**While our findings are similar to Oppong et al. (2011),^16^ we do not fully agree with their conclusion that choice of value set does not matter for cost-utility outcomes.**

- To strengthen its policy relevance, I would suggest adding the European policy context and broader European HTA landscape. The recent EU HTA regulation has a direct implication for how pooled data should be handled.

*Authors’ response*

*We have added the following sentence on the EU HTA landscape to the second paragraph in the implications section:*

**Health economic evaluations will likely continue to require localization, as these results are generally not considered transferable between countries and are therefore excluded from the European Union’s joint clinical assessment framework of health technologies.^70^**

^(…)^

**70. Johns A, Andrade A, Johal S, Ryan J. EU Joint Clinical Assessment: A Framework for Optimising Use with Cost-Effectiveness Decision-Making. *Journal of Market Access & Health Policy.* 2025 Oct 9;13(4):52.**

- Missing data in CONVINCE is concerningly high. I wonder if the authors have some possible explanations for that.

*Authors’ response*

*Unfortunately, while the overall response in the trial for PROs was rather good, the EQ-5D, iMCQ and iPCQ questionnaires were collected on paper only (whereas for other PROs we had digital collection too) and this led to lower response.*

*However, since we imputed (predicted) the missing values of the EQ-5D domains and used those values to estimate the impact of choice of value set, the rate of missing values are unlikely to impact the conclusion of this methodological research.*

- Lines 144-147, it is quite difficult to follow what is being imputed/calculated, I assume mainly due to the possibility of different terminologies being used across fields. It would be helpful if the authors consider using some mathematical formulas for clarity, which can then be put in the appendix.

*Authors’ response*

We believe this is indeed due to terminology differences across fields. In this context, multiple imputation is a specific statistical technique used to predict missing values. The predicted values are based on the distribution of the observed data of this variable, as well as the values of other ‘predictor variables’ that are not missing. Several distinct, completed datasets are created where the ‘known’ values are the same, and the imputed/predicted values differ. Using Rubin’s Rules, point estimates and uncertainty of a variable are based on the pooled/combined outcomes of the different imputed datasets, using both the variability of the ‘known’ values, as well as the imputed/predicted values. As this is a very common technique in our field, is a recommended technique in the Dutch national guideline for health economic evaluations, and in-depth references are provided (see references below), we believe that it is not necessary to add additional explanation with mathematical formulas in the appendix. However, we did clarify the methodology in the following way:

Missing values were **predicted using** multiple imputation (m = 20),^38^ applying the R mice package (see Supplementary Table 2 for predictors).^39^ The imputed datasets were analyzed using Rubin’s rules**, a method that combines (pools) results from multiple imputed datasets and adjusts the standard error to reflect the uncertainty introduced by the missing data.**^40^

Additional references:

- Burton, A., Billingham, L. J., & Bryan, S. (2007). Cost-effectiveness in clinical trials: using multiple imputation to deal with incomplete cost data. *Clinical trials*, *4*(2), 154-161.
- Dutch guideline: <https://english.zorginstituutnederland.nl/documents/2024/01/16/guideline-for-economic-evaluations-in-healthcare>, with academic reference: Geuzinge, H. A., El Alili, M., Enzing, J. J., Huis, L. M., Knies, S., & de Wit, G. A. (2025). The new Dutch guideline for economic evaluations in healthcare: taking the societal perspective to the next level. *Value in Health*, *28*(6), 930-935.

In-text references:

38. Mutubuki EN, El Alili M, Bosmans JE, et al. The statistical approach in trial-based economic evaluations matters: get your statistics together! *BMC Health Serv Res*. 2021;21:475. [https://doi.org/10.1186/s12913-021-06513-1](https://doi.org/10.1186/s12913-021-06513-1)

39. van Buuren S, Groothuis-Oudshoorn K. mice: Multivariate imputation by chained equations in R. *J Stat Softw.* 2011;45:1–67. [https://doi.org/10.18637/jss.v045.i03](https://doi.org/10.18637/jss.v045.i03)

40. Rubin DB. Multiple imputation for nonresponse in surveys. 1987. [https://doi.org/10.1002/9780470316696](https://doi.org/10.1002/9780470316696)

- If the authors would like to keep the paper up to date, the UK value set for the EQ-5D-5L is now available (<https://doi.org/10.1016/j.jval.2026.03.008> )

*Authors’ response*

*Thank you for pointing out that a new version has been published in the last month. We have replaced the values of the rejected EQ-5D-5L version with the new value set. Of note, this resulted in similar results compared to the previous version of the EQ-5D-5L value set. We have also changed the text throughout the manuscript to reflect this change, such as the following in in methods:*

For the UK, the crosswalk version of the EQ-5D-3L value set was applied using the index values at the EuroQol website,^52,53^ following the UK national guideline.^54^ **A new value set for the EQ-5D-5L has been published in March 2026, which we have included as** additional analysis.^55^ **While it has been proposed that this new EQ-5D-5L value set will replace the EQ-5D-3L cross-walk version as the preferred value set in the UK, this is not yet the case at the time of writing.^56^ Therefore, results for both value sets are presented.**

**55. Rowen D, Mukuria C, Bray N, Carlton J, Longworth L, Meads D, Oluboyede Y, O’Neill C, Yang Y. A UK value set for the EQ-5D-5L. *Value in Health*. 2026 Mar 24.** [**https://doi.org/10.1016/j.jval.2026.03.008**](https://doi.org/10.1016/j.jval.2026.03.008)

**56. National Institute for Health and Care Excellence (NICE). Have your say on NICE’s adoption of the new EQ-5D-5L value set (updated 2026 Apr 15). Available from:** [**https://www.nice.org.uk/news/articles/have-your-say-on-nices-adoption-of-the-new-eq-5d-5l-value-set. Accessed April 17**](https://www.nice.org.uk/news/articles/have-your-say-on-nices-adoption-of-the-new-eq-5d-5l-value-set.%20Accessed%20April%2017)**, 2026.**
